# Assessing the Prognostic Value of the ChOLE Classification in Predicting the Severity of Acquired Cholesteatoma

**DOI:** 10.1097/MAO.0000000000003501

**Published:** 2022-02-15

**Authors:** Maura C. Eggink, Maarten J. F. de Wolf, Fenna A. Ebbens, Frederik G. Dikkers, Erik van Spronsen

**Affiliations:** Department of Otorhinolaryngology, Amsterdam UMC, location Academic Medical Center, University of Amsterdam, The Netherlands

**Keywords:** Adverse events, ChOLE, Cholesteatoma, Classification, Recurrent disease, Residual disease, Retrospective studies, Staging

## Abstract

To assess the prognostic value of the ChOLE classification in predicting the severity of acquired cholesteatoma.

**Method:**

A retrospective chart review of patients undergoing primary cholesteatoma surgery in our tertiary referral center. The primary outcome measures were analyzed in three groups of follow up (FU): residual cholesteatoma in group *A*, FU > 52 weeks after last-look surgery or MRI-DWI; recurrent cholesteatoma in group *B*, FU > 52 weeks after last outpatient visit; and adverse events (AE) in group *C*, FU > 12 weeks after surgery. Cholesteatomata were staged according to the ChOLE classification. Kaplan–Meier curves were used to determine the prognostic value of the classification in predicting cholesteatoma severity, while correcting for FU.

**Results:**

No significant differences were observed between the various stages of the ChOLE classification and residual or recurrent cholesteatoma rate, nor the occurrence of AE. Cholesteatoma extension to the sinus tympani or widespread in the mastoid, as well as absence of the stapes superstructure were predictive of residual disease. Sclerotic mastoids had a lower risk of residual disease than mastoids with good or poor pneumatization and ventilation. Poorly ventilated and poorly pneumatized mastoids were associated with increased risk of recurrence. Widespread cholesteatoma in the mastoid as well as presence of preoperative extracranial complications were correlated with an increased risk of AE.

**Conclusion:**

The ChOLE classification does not predict residual nor recurrent disease, nor the occurrence of AE, in our study population. Risk factors for severe cholesteatoma were identified, potentially useful for the development of future classifications.

Chronic otitis media with cholesteatoma formation is a common otologic disease, requiring surgery in majority of cases and still causing severe intracranial complications if not treated effectively (1). The primary goal of cholesteatoma surgery is to eradicate disease and prevent recidivism, while secondary outcome measures include creating a dry ear and maintaining or improving hearing (2,3). A standardized classification of cholesteatoma is necessary to evaluate different surgical techniques based on the before-mentioned outcome measures. Over the past decades, multiple studies have proposed a classification of cholesteatoma, based on cholesteatoma extension, localization or origin, pathophysiology and more (4–11). To date however, no consensus has been reached (12).

In 2018, the ChOLE classification was introduced by Linder et al. (13). This system includes cholesteatoma extension (Ch), postoperative ossicular chain status (O), life-threatening complications (L) and Eustachian tube function (E). Since the introduction, only one study has been published on the effectiveness of this classification in predicting cholesteatoma severity (14). This study did not find any association between the ChOLE stage and cholesteatoma recidivism nor surgical complications.

Classification of cholesteatoma can be of great clinical value if it aids an otologic surgeon in determining treatment strategy. A classification should therefore be predictive of cholesteatoma severity, further defined as the occurrence of residual and recurrent disease and the occurrence of adverse events (AE). In this study, we evaluate the prognostic value of the ChOLE classification on these primary outcome measures.

## MATERIALS AND METHODS

### Participants

A retrospective chart analysis was done of all patients undergoing primary cholesteatoma surgery between January 1, 2004 and October 1, 2019 in a tertiary otologic referral center. The following data were collected: age, unilateral or bilateral disease, type of surgery, follow up (FU), occurrence of residual and recurrent disease, and occurrence of AE. Extension of cholesteatoma (“Ch”), ossicular chain status (“O”), presence of life-threatening complications (“L”), and mastoid pneumatization and ventilation (“E”) were defined retrospectively according to the ChOLE classification (13). All cholesteatomata were staged using the online ChOLE application (stages I–III) (15). When no CT was available, cholesteatoma were classified as “Ex” and excluded from respective analyses.

Participants were categorized in three groups based on FU: group *A*, studying residual cholesteatoma, FU > 52 weeks since last-look surgery or MRI-DWI upon primary surgery; group *B*, studying recurrent disease, FU > 52 weeks since last outpatient visit upon primary surgery; and group *C*, studying AE, FU > 12 weeks since last outpatient visit upon primary surgery. Based on this categorization, patients could be placed in more than one group.

### Types of Surgery

Cholesteatoma surgery was classified into six types of surgery (Table 1). The corresponding SAMEO-ATO classifications are noted (16).

**TABLE 1:** Types of surgery

Surgery type	SAMEO-ATO
Transcanal procedure: retro-auricular, endaural and total endoscopic (TCA)	S1A1–4MxExOx
Canal wall up procedure (CWU)	S1A4M1a-2bExOx; S1A4M1a+2aExOx; S1A4M1b+2aExOx
Canal wall up procedure with obliteration of the epitympanic and mastoid areas (CWUO)	S1A4M1a-2bExO2; S1A4M1a+2aExO2; S1A4M1b+2aExO2
Canal wall down procedure (CWD)	S1A4M2cExOx
Canal wall down procedure with subsequent reconstruction of the posterior canal wall and obliteration of the mastoid cavity (CWD-CWR)	S1A4M2cE1-2O2
Subtotal petrosectomy with blind sac closure (STP)	S1A4M3a-bExO2

Definition of types of surgery according to the SAMEO-ATO classification (16), a framework for categorization of tympanomastoid surgery based on stage of surgery (S), approach (A), mastoidectomy (M), external ear canal reconstruction (E), obliteration of mastoid cavity (O), access to middle ear (A), tympanic membrane (T) and ossicular chain (O).

### Adverse Events

Following Clavien et al (17), AE were classified into different grades (Table 2).

**TABLE 2:** Classification of adverse events of otosurgical interventions according to Clavien et al (17)

Classification	Definition	Adverse events
Grade I	Not life-threatening, no extension of hospitalization, no lasting disability	Transient postoperative vertigo; postoperative scar issues; postoperative transient facial palsy with complete recovery and preoperative iatrogenic defects of the mastoid borders, not needing any intervention
Grade II	Potentially life-threatening, no residual disability, with or without invasive procedures	Postoperative wound infection; postoperative inclusion cholesteatoma in tympanic membrane or external auditory canal; preoperative dural defects or intra-operative CSF leak requiring closure; prolonged healing period, more than 12 weeks; postoperative bleeding and hematoma requiring intervention; tympanic membrane perforation and postoperative persistent drainage
Grade III	Residual disability or persistence of life-threatening conditions	Postoperative iatrogenic sensorineural hearing loss
Grade IV	Death as result of complications	

### Statistical Analysis

Statistical analyses were performed using SPSS 27.0 (Chicago, IL). Data are expressed as number (%) and median [IQR]. Normal distribution of data was tested with a Shapiro–Wilk test. To correct for FU, Kaplan–Meier survival curves and corresponding log-rank analyses were performed to evaluate the prognostic value of the ChOLE classification on residual and recurrent disease. Only the first event of recurrent or residual disease was included. Cox regression was applied for continuous variables. Only when overall analyses of categories were significant, sub analyses were performed in which both general categories and sub categories were tested (i.e., “Ch1” as well as “Ch1a” and “Ch1b”). Chi-square and Fisher's exact tests were used to determine correlations between the ChOLE classification and AE. Sub analyses were performed to identify possible factors independently influencing outcome parameters, for example surgery type. Analyses were repeated while excluding paired ears, to rule out possible confounders (i.e., bilateral cholesteatoma). A significance level of *p* < 0.05 was used.

## RESULTS

### Participants

A total of 404 patients were eligible to enroll in this study based on the inclusion criteria. This corresponded to 440 ears, as 36 patients underwent bilateral primary surgery in our center. The clinicopathologic characteristics are presented in Table 3. A median age of primary surgery of 31.0 years (range: 3–90 years) at time of primary surgery was observed. Most patients underwent canal wall up surgery (CWU), followed by canal wall up surgery with obliteration (CWUO). In the majority of cases the cholesteatoma had extended into the middle ear, attic and antrum, corresponding to “Ch2.” Due to cholesteatoma extension or preoperative removal for adequate exposition, the ossicular chain of most patients only consisted of the malleus (with or without malleus head) and stapes postoperatively (“O1”). Overall, ChOLE stage II was highly predominant.

**TABLE 3:** Clinicopathologic characteristics

n = 440	
Ears	
Age	31.0 [38]
Surgery	
Type	
CWU	253 (57.5%)
CWUO	104 (23.6%)
TCA	43 (9.8%)
CWD	25 (5.7%)
STP	10 (2.3%)
CWD-CWR	5 (1.1%)
Cholesteatoma	
Extension	
Ch1	56 (12.7%)
Ch2	264 (60.0%)
Ch3	96 (21.8%)
Ch4	24 (5.5%)
Ossicular chain status	
O0	36 (8.2%)
O1	278 (63.2%)
O2	93 (21.1%)
O3	29 (6.6%)
O4	4 (0.9%)
Life threatening complications	
L0	387 (87.9%)
L2	51 (11.6%)
L4	2 (0.5%)
Ventilation and mastoid pneumatization	
Ex	16 (3.7%)
E0	85 (19.3%)
E1	228 (51.8%)
E2	111 (25.2%)
ChOLE	
Stage I	85 (19.3%)
Stage II	326 (74.1%)
Stage III	13 (3.0%)
Unclassified	16 (3.6%)
Recidivism	
Residual cholesteatoma	88 (27.8%)
Recurrent cholesteatoma	112 (30.4%)
Adverse events	
Total	147 (34.3%)
Grade I	84 (19.6%)
Grade II	70 (16.4%)
Grade III	11 (2.6%)
Grade IV	0 (0%)

Numbers correspond to ears with percentages rounded to the nearest tenth in parentheses, excluding age where median is given in years and interquartile range in brackets. CWD, canal wall down procedure; CWD-CWR, canal wall down mastoidectomy with subsequent reconstruction of the posterior canal wall and obliteration of the mastoid cavity; CWU indicates canal wall up procedure; CWUO, canal wall up procedure with obliteration of the epitympanic and mastoid areas; STP, subtotal petrosectomy with blind sac closure; TCA, transcanal approach. Residual disease was identified with a minimum FU of 1 year comprising of MRI-DWI or last-look surgery. Recurrent disease was diagnosed with a minimum FU of 1 year comprising of otoscopic evaluation at the outpatient clinic. Adverse events were detected with a minimum FU of 12 weeks comprising of otoscopic evaluation at the outpatient clinic. Stages of adverse events presented according to Clavien et al (17).

Group *A* included 317 ears, in which 88 residual cholesteatomata were found (27.8%). Group *B* consisted of 368 ears, of which 112 ears (30.4%) had recurrent cholesteatoma. Group *C* included 428 ears, as 12 ears were lost to otoscopic FU. In this group, in 147 ears one or more AE was observed (34.3%), mainly presenting with one pre- or postoperative AE (79.6%). The most common AE was preoperative iatrogenic defect of mastoid borders, not needing any intervention (grade I, 69 ears, 16.1%), followed by postoperative wound infection (grade II, 19 ears, 4.4%) and prolonged healing of the wound longer than 12 weeks (grade II, 18 ears, 4.2%) (Table 4).

**TABLE 4:** Occurrence of adverse events and corresponding grade according to Clavien et al. (17)

n = 428		
Adverse event	Grade	N (% total ears)
Preoperative iatrogenic defect of mastoid borders, not needing any intervention	I	69 (16.1%)
Postoperative wound infection	II	19 (4.4%)
Prolonged healing of the wound (>12 wk)	II	18 (4.2%)
Inclusion cholesteatoma in tympanic membrane or external auditory canal	II	13 (3.0%)
Tympanic membrane perforation	II	13 (3.0%)
Transient postoperative vertigo	I	11 (2.6%)
Postoperative sensorineural hearing loss	III	11 (2.6%)
Postoperative scar issues	I	10 (2.3%)
Preoperative dural defect or intra-operative CSF leak	II	6 (1.4%)
Postoperative persistent drainage	II	6 (1.4%)
Postoperative transient facial palsy with complete recovery	I	5 (1.2%)
Postoperative bleeding and hematoma requiring intervention	II	4 (0.9%)

### Effects of ChOLE Staging on Residual Cholesteatoma (Group A), Recurrent Cholesteatoma (Group B), and AE (Group C)

#### Group A: Residual Cholesteatoma Rate

**FIG. 1:**
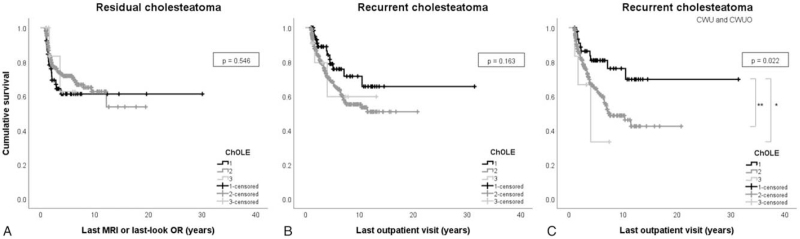
Kaplan–Meier curves and *p-*value of corresponding Log-rank analyses showing *A*, no significant correlation between ChOLE stage and residual cholesteatoma rate; *B*, no significant correlation between ChOLE stage and recurrent cholesteatoma rate and *C*, a significant correlation between ChOLE stage and recurrent cholesteatoma rate for selectively CWU and CWUO patients. ^∗^*p* < 0.05; ^∗∗^*p* < 0.01.

No significant differences were found between the various stages of the ChOLE classification overall and the residual cholesteatoma rate (Table 5, Fig. 1A). The total score of the classification was also not significant.

**TABLE 5:** Association between ChOLE classification and primary outcome measures, corrected for follow up

	Recidivism (*p*)		
ChOLE	Residual	Recurrent	Adverse events (*p*)
Stage	0.546	0.163	0.242
Total score	0.736	0.758	**<0.05**
Cholesteatoma extension (Ch)	**<0.01**	0.141	**<0.05**
Ossicular chain status (O)	**<0.05**	0.661	0.624
Life threatening complications (L)	0.194	0.776	**<0.05**
Mastoid pneumatization (E)	**<0.01**	**<0.01**	0.781

According to the ChOLE classification: cholesteatoma extension was categorized as “Ch1, 2, 3 and 4”; ossicular chain status was categorized as “O0, 1, 2, 3 and 4”; life-threatening complications were categorized as “L0, 2 and 4”; mastoid pneumatization was categorized as “E0, 1 and 2.” Residual disease was identified with a minimum FU of 1 year comprising of MRI-DWI or last-look surgery. Recurrent disease was diagnosed with a minimum FU of 1 year comprising of otoscopic evaluation at the outpatient clinic. For residual and recurrent disease *p*-values of log-rank analyses are presented for categorical variables and *p-*values of cox regression analyses are shown for continuous variable “total score”. Adverse events were detected with a minimum FU of 12 weeks comprising of otoscopic evaluation at the outpatient clinic. For adverse events *p*-values of chi-square analyses are shown for categorical values. In bold *p-*values are presented that have reached significance level *p* < 0.05, rounded to the nearest hundredth.

#### Group B: Recurrent Cholesteatoma Rate

No significant differences were found between the various stages of the ChOLE classification overall and the recurrent cholesteatoma rate (Table 5, Fig. 1B). Again, the total score of the classification was not significant. Also, no significant differences were observed between the ChOLE classification and recidivisms as a whole.

#### Group C: Adverse Effects

No significant difference between the ChOLE stage and AE occurrence was found (Table 5). Grade III AE (sensorineural hearing loss) was not correlated with a higher ChOLE stage. A higher total score was correlated with a higher chance of AE.

### Sub Analyses of ChOLE Classification Influencing Residual Cholesteatoma (Group A), Recurrent Cholesteatoma (Group B) and AE (Group C)

#### Group A: Residual Cholesteatoma Rate

Cholesteatoma extension significantly influenced residual rate (Table 5). Sub analyses showed specifically “Ch3” had a significantly higher risk of residual disease than “Ch2” (*p* < 0.01). This effect was also observed when excluding all TCA surgeries (*p* < 0.01). When further subdividing extension of disease, localization of cholesteatoma in the sinus tympani led to additional risk of residual disease as “Ch1b” had a higher risk than “Ch1a,” “Ch2a” and “Ch2b” (*p* < 0.01, *p* < 0.05, and *p* < 0.05, respectively). Extensive spread of cholesteatoma in the mastoid cavity beyond the lateral canal and/or extensive canal wall destruction was also a risk factor, as “Ch3” had a higher risk of residual disease than “Ch1a,” “Ch2a,” and “Ch2b” (*p* *<* 0.05 for all analyses). Supra- or infralabyrinthine extension (“Ch4a”) did not lead to a higher risk of residual disease compared to almost all other categories of extension (“Ch1b” to “Ch3”). Also, petrous bone cholesteatoma (“Ch4b”) did not have a higher risk of residual disease than any of the other categories. Choice of surgical approach was not significantly different for supra- or infralabyrinthine cholesteatoma (“Ch4a”), as well as apical cholesteatoma (“Ch4b”), compared to other cholesteatoma (supplementary Table 1). Pediatric patients had a higher residual rate of cholesteatoma than adult patients (*p* < 0.01). This could not be evidently correlated to a greater extension of pediatric cholesteatoma, as both “Ch1” and “Ch4” cholesteatoma were significantly more common in the pediatric group (*p* < 0.001 and *p* < 0.01, respectively).

Ossicular chain status was correlated with residual rate: sub analyses showed “O2” had a significantly higher risk of residual disease than “O1” (*p* < 0.01). Absence of the stapes superstructure (“O2,” “O3b,” and “O4b”) was correlated with cholesteatoma localization in the sinus tympani (“Ch1b,” “Ch2b,” “Ch3,” and “Ch4a”) (*p* < 0.01). Life-threatening complications did not have any effect on residual disease.

Mastoid pneumatization did influence residual disease as a sclerotic mastoid (“E2”) had a significantly lower risk of residual disease compared to both poorly (“E1”) and well-pneumatized and well-ventilated mastoids (“E0”) (*p* < 0.01 for both analyses). This effect could not be explained by choice of surgical approach as sub analyses showed no relevant differences in the distribution of this effect across the various surgical techniques (supplementary Table 1).

#### Group B: Recurrent Cholesteatoma Rate

Cholesteatoma extension, ossicular chain status and life-threatening complications did not influence recurrent disease (Table 5). Mastoid pneumatization did influence recurrence of cholesteatoma; a poorly pneumatized and ventilated mastoid (“E1”) had a higher risk of recurrent disease than a mastoid with good pneumatization and ventilation (“E0”) or a sclerotic mastoid (“E2”) (*p* *<* 0.01 and *p* < 0.05, respectively). As there were no relevant differences between choice of surgical approach and mastoid pneumatization, surgical approach was not a confounder in this analysis (supplementary Table 1).

#### Group C: Adverse Events

Cholesteatoma extension did influence the chance of occurrence of AE (Table 5), where “Ch3” had a higher risk of AE occurrence than “Ch2” (*p* < 0.01). Ossicular chain status was not correlated with occurrence of AE. Life-threatening complications were correlated with AE, as ears with extracranial complications had a significantly higher chance of AE than ears without extracranial complications (*p* < 0.05). More specifically, there was increased risk of sensorineural hearing loss (*p* < 0.05), persistent drainage (*p* < 0.01), postoperative vertigo (*p* < 0.05) and postoperative wound infection (*p* < 0.05). It is possible choice of surgical approach plays a role as CWD and CWD-CWR were preferred when extracranial complications were present (*p* *<* 0.01 and *p* < 0.001, respectively) and CWD was associated with a higher rate of AE (*p* < 0.05), while STP was preferred in patients with intracranial complications (*p* < 0.05).

### Effects of ChOLE Stage on Residual Cholesteatoma (Group A), Recurrent Cholesteatoma (Group B) and AE (Group C), Stratified by Type of Surgery

The ChOLE stage was partially predictive of recurrent disease in selectively CWU patients, and in CWU and CWUO patients combined (Fig. 1C), as both stage II and III had a significantly higher risk of recurrence than stage I (*p* < 0.01 and *p* < 0.05, respectively). Staging did not lead to significant differences in recurrence after TCA, CWD, CWD-CWR, and STP surgeries. For residual cholesteatoma and AE, ChOLE stage did not differ significantly between any of the surgical approaches.

Choice of CWU, CWD, CWD-CWR, and STP in our population was not influenced by cholesteatoma extension. Only “Ch1” cholesteatoma were preferably operated with TCA (*p* < 0.001) and “Ch3” cholesteatoma were preferably operated with CWUO (*p* < 0.05) (supplementary Table 1).

There was no significant correlation between poorly pneumatized or sclerotic mastoids and choice of CWD, CWU, CWUO, or CWD-CWR (supplementary Table 1). Significantly more sclerotic mastoids were found in the STP group (*p* < 0.01) and significantly more well-pneumatized and well-ventilated mastoids were found in the TCA group (*p* < 0.001). As the residual and recurrence rates of cholesteatoma in these groups do not significantly differ from the other surgical approaches, this is not expected to be a confounder in analyses.

Rates of residual and recurrent disease, as well as occurrence of AE, stratified per OR type and corrected for FU, are presented in supplementary Table 1 and in previous work (18).

No significant differences in results were found when correcting for paired ears. Ears with missing data were excluded from corresponding analyses: 16 cases could not be staged retrospectively as CT imaging, necessary to determine the extent of mastoid pneumatization, was missing.

## DISCUSSION

The efforts of Linder et al. to propose a novel cholesteatoma staging system, the ChOLE classification, should be highly appreciated, as world-wide consensus on an adequate system has yet to be reached. It is a well-defined classification, accompanied by an intuitive online application for easy staging. It has a clear purpose: “to evaluate and compare the outcome of various surgical approaches and philosophies” (13). A classification is of clinical value if it accurately defines disease severity and correlates with different prognoses. It is only then, that staging may have consequences when determining the type of surgical approach, as a higher stage could warrant more aggressive treatment and possibly more intensive FU (19).

We tested the efforts of Linder et al. in our relatively large population, which yielded similar rates of residual and recurrent cholesteatoma as previous reports (20,21). Unfortunately, our study was unable to demonstrate significant differences between recidivism and ChOLE stage in those ears with a minimum FU of 1 year and correcting for FU by using Kaplan–Meier curves. It also demonstrated that the ChOLE stage does not predict the occurrence of residual nor recurrent disease as separate entities, nor the occurrence of AE. This is in line with the only other study investigating the utility of the ChOLE classification, done by authors affiliated to the developers of the ChOLE classification, albeit with a relatively short clinical FU limited to one year FU upon surgery (14).

The ChOLE stage is partially predictive of recurrent cholesteatoma in CWU patients, as well as in CWU and CWUO patients combined. Previously, other studies have shown partial effects of the JOS, EAONO-JOS, and STAMCO classifications in specific surgical approaches (18,20). The value of a classification that only holds true for one or two specific surgical approaches is limited as it is unlikely to have implications in determining treatment strategies. By identifying which subsets of the classification influence the occurrence of residual or recurrent cholesteatoma, or AE, we aim to suggest improvements of the ChOLE classification thereby possibly improving the prognostic value of the ChOLE classification.

### Cholesteatoma Extension, “Ch”

The ChOLE classification focuses on cholesteatoma extension from the tympanic cavity, similar to previous efforts proposing a classification (8,9). Our findings suggest two specific “high risk areas” for residual disease, rather than a gradual increase in risk as size increases.

Firstly, the EAONO-JOS and STAMCO classifications, which focus on cholesteatoma localization rather than extension, have defined the sinus tympani as a “difficult access site” (22,23). This study also identifies isolated extension to the sinus tympani to be a risk factor for residual cholesteatoma. This result is in line with our previous work on staging cholesteatoma using the JOS, EAONO-JOS, and STAMCO classifications (18). This finding implies that possibly a more rigorous surgical approach is used when there is extensive spread of cholesteatoma beyond the tympanic cavity, preventing cholesteatoma in the sinus tympani to be overlooked. Also, it emphasizes the importance of thorough inspection of the sinus tympani in every surgical approach. The complementary use of a rigid endoscope can expose any remnants of cholesteatoma in the sinus tympani after microscopic surgery, possibly decreasing the risk of residual disease (24,25).

Secondly, widespread of the cholesteatoma in the mastoid cavity beyond the lateral canal and/or extensive destruction of the external ear canal, was identified as a second “high risk area” for residual disease, as well as the occurrence of AE. Localization of disease in the mastoid has previously been linked with disease recidivism as a whole (26). It might be expected that localization of disease in the mastoid cavity beyond the lateral canal would increase the risk of residual disease, or recidivism as a whole, as TCA yields little or no exposition of the mastoid. Remarkably, this could not be confirmed by our study. This can be explained due to the fact that only a limited number of ears (16 ears with a minimum FU of 1 year) could be included in this sub analyses and there was a relatively high recidivism rate, regardless of cholesteatoma spread.

Absolute size of cholesteatoma is not an adequate predictor of cholesteatoma severity as there was no increase in residual disease with supra- or infra-labyrinthine spread, regardless of surgical approach. Furthermore, cholesteatoma recurrence was not influenced by cholesteatoma extension overall, while cholesteatoma extension did not differ relevantly per OR type. These findings are in line with findings of Britze et al., who did not find an association between cholesteatoma limited to the tympanic cavity and reduction of recidivism as a whole (26). Our study also implies petrous cholesteatoma do not behave more aggressively as severity does not significantly differ, regardless of surgical approach. Furthermore, no gradual increase in the occurrence of AE was found as cholesteatoma extension increased. We therefore advocate cholesteatoma severity to be based on extension to “high risk areas” rather than absolute size or type.

### Ossicular Chain Status, “O”

Our study shows that the status of the ossicular chain could potentially reflect cholesteatoma severity, albeit partially. This is consistent with previous reports, in which erosion of the incus and stapes have independently been correlated with disease recidivism as a whole (26). In our study, the association between absence of the stapes superstructure and higher risk of residual disease can be explained by expected localization of cholesteatoma in the “high risk area” sinus tympani, due to the relatively close proximity to the stapes in the mesotympanum. In our center the goal is to preserve the stapes superstructure at all times, due to risk of dislocation of the footplate. Therefore, if only the stapes footplate remains preoperatively, disease must have been localized around the stapes and classification “O2” can directly imply destruction of the stapes superstructure due to cholesteatoma extension. However, as surgical approach of the stapes may vary world-wide, the value of preoperative ossicular chain status in predicting cholesteatoma severity before completing OCR, is doubtful.

### Complications, “L”

The presence of complications is not predictive of disease recidivism. However, presence of extra- or intracranial complications does alter an otologic surgeon's preference of surgical approach. Also, the presence of extracranial complications could suggest more severe cholesteatoma as the presence of extracranial complications was associated with the presence of postoperative AE. This could justify an altered clinical FU, to anticipate possible occurrence of AE, for instance specifically after CWD surgery.

### Mastoid Pneumatization and Ventilation, “E”

Previous classifications have included the development and extent of pneumatization and ventilation of mastoid cells, without it leading to a higher stage of cholesteatoma (27). The ChOLE classification does include mastoid pneumatization and ventilation in its staging system and our findings indeed suggest that the development of mastoid cells is predictive of cholesteatoma severity. A poorly pneumatized and poorly ventilated mastoid (i.e., diploic mastoid) was associated with a higher rate of recurrent cholesteatoma, regardless of surgery type. This suggests that suppressed mastoid cell development contributes to, or is a symptom of, the pathogenesis of the cholesteatoma. This is in line with previous reports showing mastoid air cell development to be suppressed more severely in chronic otitis media with acquired cholesteatoma than in chronic otitis media without cholesteatoma (28). Mastoid pneumatization ipsilateral to cholesteatoma has also been shown to be underdeveloped in comparison to healthy contralateral ears. Altogether this suggests that chronic inflammation or the presence of cholesteatoma matrix suppresses mastoid pneumatization, as previously suggested in the environmental pneumatization theory (29,30).

Interestingly, sclerotic mastoids, with no pneumatization at all and limited ventilation, did not have significantly higher risk of recurrent cholesteatoma when compared to poorly pneumatized and poorly ventilated (diploic) mastoids, introducing the possibility of a tipping point in pathology. The authors therefore hypothesize that if the mastoid is completely dense, no sick mucosa is present to maintain inflammation and/or a negative middle-ear pressure, resulting in lower recurrence rates. This could warrant a more aggressive surgical approach including obliteration of the epitympanum and mastoid bowl, aiming to eradicate sick mucosa and drill out remaining poorly ventilated mastoid cell tracts.

Sclerotic mastoids were associated with a lower risk of residual disease than mastoids with good and poor pneumatization and ventilation, also regardless of surgical approach. Surgically removing disease from widely pneumatized mastoid cells is more challenging, especially in noncyst like cholesteatomata that easily infiltrate remaining air cell tracts, than removing it from dense bone. The combination of a poorly pneumatized mastoid and widespread cholesteatoma into the “high risk area” mastoid could demand an even more meticulous approach to eradicating disease during surgery.

Apart from preventing residual and recurrent disease, cholesteatoma surgery aims to create a hygienic and waterproof ear, while maintaining or improving hearing. In our study, we did not look into the value of the ChOLE classification in predicting these secondary outcome measures. The presence of preoperative extracranial complications was associated with more postoperative AE in the short-term. The classification could be used to counsel patients on a potentially more challenging recovery after surgery, especially after CWD. More intensive outpatient clinic appointments could be justified to follow-up on wound healing, postoperative vertigo or sensorineural hearing loss and prophylactic antibiotics could be prescribed to prevent postoperative wound infections. In a recent study, the health-related quality of life as measured by the Zurich Chronic Middle Ear inventory, was not associated with ChOLE staging in the long-term (31). On the contrary, hearing outcomes postoperatively were associated with ossicular chain status before completing OCR (14,31). The ChOLE stage could therefore evaluate functional outcomes of hearing after cholesteatoma surgery. However, we question the added benefit of staging for this purpose, as it is not applicable in counseling patients in the outpatient clinic before surgery and thus has limited clinical consequences.

A limitation of this study could be the fact that ChOLE stage II cholesteatoma were overrepresented in our population, diminishing the discriminative ability of the classification. A previous study confirmed preponderance of ChOLE stage II cholesteatoma in a population with comparable state of health care (31). Furthermore, the application of the ChOLE classification in a retrospective chart analysis could decrease the accuracy of the classification. However, Linder et al. developed an intuitive online application for straight-forward postoperative classification. Also, recent research has demonstrated retrospective classification is possible, but interrater agreement for certain areas of the ChOLE classification was found to be limited (32). Intrarater agreement of the ChOLE classification has not been analyzed. In our study, one single otologic surgeon was involved in classifying all cholesteatoma and therefore interrater variability cannot be a confounder. In conclusion, we deem our retrospective classification to be adequate.

A strength of the ChOLE classification is the use of a scoring system, summing up to a certain cholesteatoma stage. The classification assigns points to, for instance, extra- and intracranial complications, rather than automatically staging the cholesteatoma in the severest category when intracranial complications are present, as in the EAONO-JOS classification (22). This allows for a more balanced inclusion of factors that could play a role in cholesteatoma severity. Redefining the cut-off points for the various stages could improve the prognostic value of the classification slightly although the total amount of points assigned by the ChOLE classification was not significantly associated with recurrent or residual disease.

In our opinion, the ChOLE classification in its current form is a registration system, providing a valid basis for standard reporting of cholesteatoma. However, there is a fundamental difference between a registration system and a classification. The ChOLE staging does not have the power to differentiate between less aggressive and more severe cholesteatoma. Therefore, it has no consequences for choice of appropriate treatment strategy and FU scheme and therefore does not measure up to our previously described definition of a classification with clinical value. The current classification could be tested in subgroups stratified by SAMEO-ATO classification of tympanomastoid surgery, however the clinical value of such a classification is also limited in our opinion. In the future, a classification could be considered including only previously identified risk factors for residual and recurrent cholesteatoma as well as AE. We challenge the need to categorize all aspects of cholesteatoma, if these aspects have limited influence on the primary outcome measures. Such a classification could include the risk factors identified in this study: localization of cholesteatoma in the “high risk area” sinus tympani or widespread into the mastoid cavity, and poorly pneumatized and ventilated mastoid cells. Additional risk factors such as age, previously reported in other studies and also confirmed in our data, could also be included (18,26). Once this classification has been developed, it should be tested in another population, using data corrected for FU. Finally, a prognostic multi-center study should validate the prognostic value of a potential classification in predicting severity of acquired cholesteatoma.

## CONCLUSION

The ChOLE classification does not predict residual and recurrent cholesteatoma, nor the occurrence of AE in our population. Risk factors for severe cholesteatoma were identified: localization of cholesteatoma in the sinus tympani and widespread cholesteatoma into the mastoid cavity, as well as poorly pneumatized and ventilated mastoid cells. In the future, a classification could be developed including these specific risk factors, potentially defining stages of cholesteatoma with different prognoses, benefitting from different treatment strategies.

## Supplementary Material
