# Supplementary material for: Assessing the Prognostic Value of the ChOLE Classification in Predicting the Severity of Acquired Cholesteatoma
Source: Otol Neurotol. 2022 Feb 15;43(4):472–80. doi: 10.1097/MAO.0000000000003501 (PMC8915987; doi:10.1097/MAO.0000000000003501)
Supplement: Supplementary file 1 [file mao-43-472-s001.docx]

Supplementary table 1:

|  |  | |  |  | |  | |  | |  |
| --- | --- | --- | --- | --- | --- | --- | --- | --- | --- | --- |
|  | CWU | CWD | | CWUO | CWD-CWR | | STP | | TCA | |
| **Cholesteatoma extension (Ch)** |  |  | |  |  | |  | |  | |
| Ch1 | 28 (50.0%) | 2 (3.6%) | | 6 (10.7%)* | 0 (0%) | | 0 (0%) | | 20 (35.7%)*** | |
| Ch2 | 162 (61.4%) | 13 (4.9%) | | 60 (22.7%) | 3 (1.1%) | | 4 (1.5%) | | 22 (8.3%) | |
| Ch3 | 48 (50.0%) | 9 (9.4%) | | 32 (33.3%)* | 2 (2.1%) | | 4 (4.2%) | | 1 (1.0%)** | |
| Ch4 | 15 (62.5%) | 1 (4.2%) | | 6 (25.0%) | 0 (0%) | | 2 (8.3%) | | 0 (0%) | |
| **Ossicular chain status (O)** |  |  | |  |  | |  | |  | |
| O0 | 20 (55.6%) | 1 (2.8%) | | 0 (0%)*** | 0 (0%) | | 0 (0%) | | 15 (41.7%)*** | |
| O1 | 152 (54.7%) | 17 (6.1%) | | 84 (30.2%)*** | 2 (0.7%) | | 1 (0.4%)*** | | 22 (7.9%) | |
| O2 | 64 (68.8%) | 5 (5.4%) | | 18 (19.4%) | 1 (1.1%) | | 0 (0%) | | 5 (5.4%) | |
| O3 | 15 (51.7%) | 2 (6.9%) | | 2 (6.9%)* | 2 (6.9%) | | 7 (24.1%)*** | | 1 (3.4%) | |
| O4 | 2 (50.0%) | 0 (0%) | | 0 (0%) | 0 (0%) | | 2 (50.0%)** | | 0 (0%) | |
| **Life threatening complications (L)** |  |  | |  |  | |  | |  | |
| L0 | 235 (60.7%)*** | 16 (4.1%)** | | 86 (22.2%) | 1 (0.3%)*** | | 6 (1.6%)* | | 43 (11.1%)* | |
| L2 | 17 (33.3%)*** | 9 (17.6%)** | | 18 (35.3%) | 4 (7.8%)*** | | 3 (5.9%) | | 0 (0%)* | |
| L4 | 1 (50.0%) | 0 (0%) | | 0 (0%) | 0 (0%) | | 1 (50.0%)* | | 0 (0%) | |
| **Ventilation and mastoid pneumatization (E)** |  |  | |  |  | |  | |  | |
| Ex | 9 (56.3%) | 3 (18.8%) | | 3 (18.8%) | 0 (0%) | | 1 (6.3%) | | 0 (0%) | |
| E0 | 47 (55.3%) | 1 (1.2%) | | 12 (14.1%) | 0 (0%) | | 0 (0%) | | 25 (29.4%)*** | |
| E1 | 139 (61.0%) | 14 (6.1%) | | 61 (26.8%) | 2 (0.9%) | | 2 (0.9%) | | 10 (4.4%)*** | |
| E2 | 58 (52.3%) | 7 (6.3%) | | 28 (25.2%) | 3 (2.7%) | | 7 (6.3%)** | | 8 (7.2%) | |
| **ChOLE** |  |  | |  |  | |  | |  | |
| Stage I | 42 (49.4) | 1 (1.2%) | | 12 (14.1%)* | 0 (0%) | | 0 (0%) | | 30 (35.3%)*** | |
| Stage II | 197 (60.4%) | 20 (6.1%) | | 88 (27.0%)** | 3 (0.9%) | | 5 (1.5%) | | 13 (4.0%)*** | |
| Stage III | 5 (38.5%) | 1 (7.7%) | | 1 (7.7%) | 2 (15.4%)** | | 4 (30.8%)*** | | 0 (0%) | |
| **Cholesteatoma severity** |  |  | |  |  | |  | |  | |
| Residual cholesteatoma | 74 (84.1%)*** | 3 (3.4%)* | | 3 (3.4%)*** | 0 (0%) | | 1 (1.1%) | | 7 (8.0%) | |
| Residual cholesteatoma rate | 32.6% | 21.4% | | 5.7% | 0% | | 16.7% | | 43.8% | |
| Recurrent cholesteatoma | 103 (92.0%)** | 2 (1.8%) | | 3 (2.7%)*** | 0 (0%) | | 0 (0%) | | 4 (3.6%) | |
| Recurrent cholesteatoma rate | 43.6% | 8.3% | | 9.6% | 0% | | 0% | | 16.7% | |
| Adverse events | 87 (59.2%) | 14 (9.5%)* | | 32 (21.8%) | 1 (0.7%) | | 6 (4.1%) | | 7 (4.8%)* | |

Number of ears noted with percentages of occurrence across surgery types in parentheses, rounded to the nearest tenth. CWU: canal wall up procedure, CWD: canal wall down procedure, CWUO: canal wall up procedure with obliteration of the mastoid, CWD-CWR: canal wall down procedure with subsequent reconstruction of the posterior canal wall and obliteration of the mastoid cavity, STP: subtotal petrosectomy with blind sac closure, TCA: transcanal procedure (retro-auricular, endaural and total endoscopic). Residual disease was identified with a minimum FU of 1 year comprising of MRI-DWI or last-look surgery. Recurrent disease was diagnosed with a minimum FU of 1 year comprising of otoscopic evaluation at the outpatient clinic. Adverse events were detected with a minimum FU of 12 weeks comprising of otoscopic evaluation at the outpatient clinic. * p<0.05; ** p<0.01; *** p<0.001
